# Impact of Indocyanine Green Angiography on Postoperative Parathyroid Function: A Propensity Score Matching Study

**DOI:** 10.3390/jcm13113038

**Published:** 2024-05-22

**Authors:** Salih N. Karahan, Safa Toprak, Burak Celik, Ibrahim H. Ozata, Defne Yigci, Mekselina Kalender, Serdar Tezelman, Orhan Agcaoglu

**Affiliations:** 1 Department of General Surgery, School of Medicine, Koc University, Istanbul 34450, Turkey; skarahan@kuh.ku.edu.tr (S.N.K.); satoprak@kuh.ku.edu.tr (S.T.); bcelik@kuh.ku.edu.tr (B.C.); iozata@ku.edu.tr (I.H.O.); dyigci21@ku.edu.tr (D.Y.); mkalender17@ku.edu.tr (M.K.); stezelman@kuh.ku.edu.tr (S.T.); 2 Department of General Surgery, American Hospital, Istanbul 34365, Turkey

**Keywords:** fluorescence imaging, indocyanine green angiography, parathyroid preservation, thyroidectomy

## Abstract

**Background**: Thyroidectomy constitutes an important portion of endocrine surgery procedures and is associated with various complications such as bleeding, recurrent laryngeal nerve injury, and postoperative hypoparathyroidsm. Effective parathyroid preservation during thyroid surgery is crucial for patient well-being, with current strategies heavily reliant on surgeon experience. Among various methods, Indocyanine Green Angiography (ICGA) offers a promising method for intraoperative assessment of parathyroid gland perfusion. **Methods**: In a retrospective study, patients undergoing bilateral thyroidectomy from January 2021 to January 2023 were analyzed, excluding those with previous thyroidectomy, parathyroid disease, or chronic kidney disease. The study compared a control group (*n* = 175) with an ICGA group (*n* = 120), using propensity score matching for statistical analysis. Matched cohorts included 120 patients in each group. The primary outcome of this study was identified as temporary postoperative hypoparathyroidism, with secondary outcomes including the rate of parathyroid reimplantation and the incidence of permanent postoperative hypoparathyroidism. **Results**: The ICGA group showed significantly more parathyroid autotransplantations (*p* < 0.01). While not statistically significant, the control group had a higher incidence of temporary postoperative hypoparathyroidism (*p* < 0.09). Rates of hypocalcemia on postoperative day 1 and permanent hypocalcemia were similar. Subgroup analysis indicated more postoperative day 1 hypoparathyroidism in the control group during central neck dissections (*p* < 0.049). **Conclusions**: Intraoperative ICGA use correlated with higher parathyroid autotransplantation and suggested reduced postoperative hypoparathyroidism. Changes in fluorescence intensity following a second ICG injection may provide an objective method to assess parathyroid perfusion. Further large-scale studies are needed to fully understand ICGA’s impact on parathyroid preservation.

## 1. Introduction

Surgeries involving the thyroid and parathyroid glands represent a significant portion of endocrine surgical procedures, with over 150,000 thyroidectomies conducted each year in the United States alone [1]. Among the various complications associated with total thyroidectomy, hypoparathyroidism stands out due to its frequency and impact on patient well-being. The incidence of temporary and permanent hypoparathyroidism was reported to be between 19% and 38% and 1% and 3%, respectively [2]. Preventing hypoparathyroidism by the preservation of the parathyroid gland during surgery is important for the patient’s quality of life, as it is associated with multiple hospital visits, longer hospital stays, the need for long-term replacement therapy and osteoporosis risk [3].

Traditional techniques for parathyroid gland preservation are limited as they are surgeon-dependent and lack the means of determining gland vascularization and perfusion by the naked eye. Moreover, parathyroid glands usually have atypical locations and are hard to differentiate from surrounding fat tissue with the naked eye [4]. A previous study by Sitges-Serra et al. has shown that between 20% to 50% of thyroidectomy patients require autotransplantation of one or more parathyroid glands, with 22% experiencing unintentional removal of these glands [5]. Thus, developing a straightforward and dependable technique to aid the intraoperative detection of parathyroid glands would be highly advantageous. To address this challenge, with the help of technological advancement, new techniques including fluorescence imaging with indocyanine green angiography (ICGA) that can assess both parathyroid gland vascularization and perfusion were developed.

Indocyanine green (ICG) fluorescence angiography (FA) represents a significant advancement in the intricate challenge of evaluating visceral perfusion, demonstrating extensive utility across a multitude of medical and surgical disciplines [6,7]. This technique involves the intravenous administration of ICG, a fluorophore dye characterized by its distinctive fluorescence when excited by near-infrared (NIR) radiation. The resultant fluorescent signal is directly proportional to blood flow, enabling surgeons to identify and rectify regions of suboptimal perfusion intraoperatively, a task that presents considerable difficulties using traditional observational methods [8]. 

Moreover, ICGA has been employed in diverse medical and surgical specialties for the assessment of perfusion, including but not limited to bowel perfusion during bowel anastomosis, evaluation of skin flap perfusion, and visualization of bile duct anatomy. It also plays a pivotal role in assessing lymphatic limb perfusion of anastomosis and in the mapping of lymphatic drainage for the diagnosis of cancer [9]. Beyond perfusion evaluation, ICG’s applications extend to the determination of cardiac output, hepatic function assessment, and facilitation of fluorescence-guided surgery (FGS). Specifically, ICG is instrumental in intraoperative localization techniques such as sentinel lymph node mapping and metastasis detection, tissue perfusion assessment for surgical resection and anastomosis, imaging of critical structures (e.g., during cholangiography), and the identification of leaks and targeted therapies [10]. The extensive range of ICG-FA applications underscores its integral role in enhancing surgical precision and optimizing patient outcomes.

However, its use in thyroidectomy to preserve and predict parathyroid function is relatively new. Recent studies have validated the applicability of indocyanine green (ICG) fluorescence imaging in detecting parathyroid glands and their vascular architecture during thyroidectomy [11,12,13,14,15]. However, there is a scarcity of data regarding the relationship between ICGA findings and their impact on postoperative parathyroid function, with conflicting results reported. Our objective is to evaluate the impact of fluorescence imaging with ICGA in preserving parathyroid glands and predicting possible postoperative parathyroid dysfunction.

## 2. Materials and Methods

This retrospective study was conducted in two tertiary care hospital endocrine surgery departments. The patient selection criteria for this study encompassed individuals aged 18 years or above who had undergone bilateral thyroid surgery, with or without central neck dissection for both benign and malignant diseases, from January 2021 to January 2023. Patients who have a previous history of thyroidectomy, parathyroid disease and chronic kidney disease were excluded. Central neck lymph node dissection, defined as Level VI lymph node dissection, involved the removal of lymph nodes within the anatomical boundaries of the hyoid bone superiorly, the suprasternal notch inferiorly, and the carotid arteries laterally. This area includes prelaryngeal, pretracheal, and paratracheal nodes. 

Fluorescent imaging using Indocyanine Green Angiography (ICGA) was incorporated into our clinical routine in January 2021, and has since been systematically applied to patients undergoing total thyroidectomy. However, its usage has been contingent upon individual patients’ insurance status and the extent to which ICGA is covered by their insurance plans. Eligible patients were divided into two cohorts: the control group, comprising patients who underwent total thyroidectomy via conventional methods, and the ICGA group, which consisted of patients who underwent total thyroidectomy utilizing fluorescent imaging with indocyanine green. Patient demographics, including age, gender, body mass index, indication for surgery, preoperative and postoperative biochemical results (calcium [Ca] and parathyroid hormone [PTH] levels), operative details (type and duration of surgery, use of ICGA, number of intraoperative detected parathyroid glands), data related with follow-up visits and morbidity status were retrieved from a database that is prospectively recorded.

**Figure 1:**
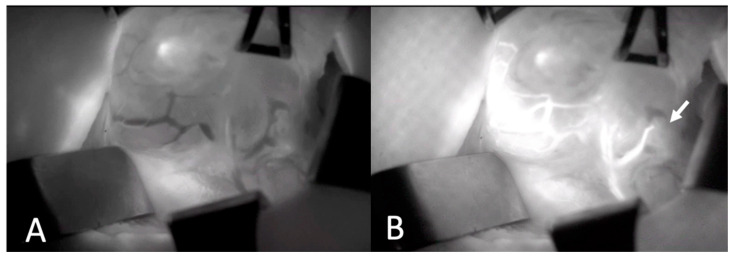
Intraoperative Images of Thyroid Gland and Parathyroid Gland Vascularization Before (**A**) and after (**B**) ICGA. Arrow depicts lower parathyroid gland with intact vascularization (ICGA: Indocyanine green angiography).

**Figure 2:**
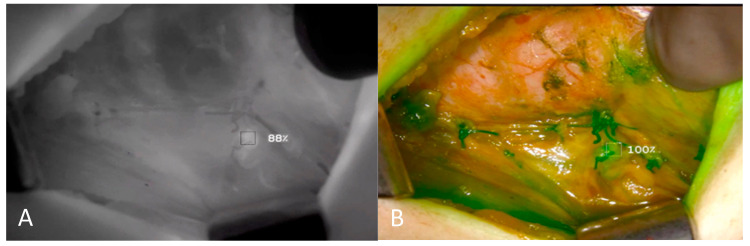
Intraoperative Images of Left Upper Parathyroid Gland. Fluorescence intensity showing perfusion of parathyroid gland is quantified and compared by contrast angiography (**A**,**B**).

Two experienced board-certified endocrine surgeons performed the operations. For all patients, the conventional subcapsular thyroidectomy approach was used. After a Kocher incision, the subcutaneous tissues and platysma were divided with electrocautery. Subplatysmal flaps were then raised inferiorly extending into the sternal notch and superiorly extending to the thyroid cartilage. The strap muscles were divided and retracted laterally. The right and left thyroid glands were mobilized laterally by blunt dissection and using electrocautery. Retracting the thyroid lobe medially, the middle thyroid vein is identified and ligated. The anterior branches of the superior thyroid artery were dissected and ligated taking care not to harm the external branch of the superior laryngeal nerve. The superior parathyroid gland was then identified by a thorough examination of the superior pole of the thyroid. Then, the inferior parathyroid gland was searched around the inferior pole. Before any dissection, the presumed parathyroid glands were seen with the naked eye. On the opposite side, a similar procedure was used to ensure that as many parathyroid glands as possible could be seen. A solution of ICG (Verdye^TM^, Diagnostic Green Ltd., Athlone, Ireland) was created by dissolving 25 mg of powdered ICG in 10 mL of distilled water. For each ICG study, 1 mL of the solution was injected, and the IV line was washed with 10 mL of saline. The vascular supply of the detected parathyroid glands was visualized using SPY-PHI camera (Stryker Corp. Kalamazoo, MI, USA) (Figure 1). All parathyroid glands identified by the SPY-PHI camera were counted and their locations were recorded. The vascular supply of each gland was noted, and dissection was continued accordingly, taking care not to harm any vascular branch supplying parathyroid glands. Thyroidectomy was then completed, and the specimen was removed en-block. An additional 1 mL of ICG was injected and under fluorescence mode, the perfusion of parathyroid glands was visualized (Figure 2). The perfusions of parathyroid glands were compared to the trachea in SPY Fluorescence mode using the fluorescence intensity measurement property. If one gland seemed to be poorly perfused, under direct visualization of the SPY-PHI camera, another 1 mL of dissolved ICG was injected, and the change in fluorescence intensity was inspected in SPY Fluorescence mode. If the gland showed less than 25% increase in fluorescence intensity, the gland was assumed to be nonviable and was excised and autotransplanted. Additionally, the removed thyroidectomy specimen was also imaged using SPY-PHI camera for any parathyroid gland. If found, they were reimplanted.

Patients were followed up in the surgical ward and were monitored for signs of hypocalcemia. On postoperative day 1, serum calcium, PTH, phosphorus, and albumin levels were measured. Post-thyroidectomy hypoparathyroidism was defined as the postoperative PTH < 15 pg/mL and postoperative hypocalcemia was defined as corrected serum Ca level < 8 mg/dL. Patients with hypoparathyroidism on day 1 were prescribed calcium and vitamin D supplements on discharge. In the control visit (POD 10–14), serum calcium and PTH levels were assessed, and hypoparathyroidism persisted after postoperative six months, it was defined as permanent. 

Statistical analysis of the results was performed using SPSS version 28.0 (IBM, Armonk, NY, USA). Continuous variables were expressed as mean ± standard deviation (SD) or median (range) based on data distribution, whereas categorical variables were presented as absolute values and percentages. Student’s *t*-test and Mann–Whitney U test were used to compare normally and non-normally distributed variables, respectively. Fisher’s exact test and chi-square test were used for the analysis of categorical variables. 

Propensity score matching with the nearest neighbor matching method was used to minimize selection bias. Fluorescence imaging and control groups were matched at a 1:1 ratio with a match tolerance of 0.2. Variables such as age, sex, indication for surgery, preoperative calcium level, BMI, and presence of central neck dissection were included in the propensity score model. After the matching, parathyroid gland autotransplantation and postoperative temporary and permanent hypoparathyroidism rates were compared between the two groups. Statistical significance was defined by a *p*-value < 0.05. 

The study was approved by the institution’s review board and ethics committee (IRB No: 2023.275.IRB1.089). This study was conducted in compliance with the 1964 Helsinki Declaration. All subjects agreed on a written informed consent before their participation. All methods were carried out according to the relevant guidelines and regulations of our institutional review board.

## 3. Results

Between January 2021 and January 2023, 295 patients underwent bilateral total thyroidectomy. In 120 patients, ICGA was used while, in 175 cases, conventional methods were used for parathyroid gland preservation. Clinicopathological, demographic, and treatment characteristics of patients before propensity score matching are shown in Table 1. The gender distribution was 75.4% female in the control group and 65.8% in the ICGA group, with a *p*-value of 0.07. Age was comparable across groups, with averages of 49.14 ± 13.36 years in the control and 49.16 ± 13.13 years in the ICGA group (*p* = 0.99). Body Mass Index (BMI) also showed no statistical difference, with values of 27.3 ± 5.26 in the control group and 26.95 ± 5.36 in the ICGA group (*p* = 0.58). Regarding the indication for surgery, 44% of the control group and 54.2% of the ICGA group were operated on for malignancy (*p* = 0.09). Preoperative serum calcium and vitamin D levels were similar between groups (*p* = 0.24 and *p* = 0.76, respectively). The frequency of central neck dissection was 42.9% in the control group and 40.8% in the ICGA group (*p* = 0.79).

After propensity score matching, 120 patients were found in each group. Clinicopathological, demographic, and treatment characteristics of patients after propensity score matching are shown in Table 2. The distribution of gender between the control and ICGA groups showed no significant difference, with 73.3% females in the control group compared to 65.8% in the ICGA group (*p* = 0.21). The average age was comparable between the control group (48.69 ± 1.2 years) and the ICGA group (49.16 ± 13.1 years) with a *p*-value of 0.78. Body Mass Index (BMI) was also similar across the groups, with 26.87 ± 5.3 in the control group and 26.95 ± 5.36 in the ICGA group (*p* = 0.91). In terms of the indication for surgery, 49.2% of the control group and 54.2% of the ICGA group underwent surgery due to malignancy, showing no significant difference (*p* = 0.43). Preoperative serum calcium and vitamin D levels were likewise comparable between the two groups, with *p*-values of 0.21 and 0.81, respectively. The rate of central neck dissection was 45% in the control group and 40.8% in the ICGA group, with no significant statistical difference observed (*p* = 0.51).

Postoperative outcomes after propensity score matching are shown in Table 3. Significant differences were observed in the rate of parathyroid gland reimplantation, with only 0.8% in the control group compared to 10% in the ICGA group, indicating a significant increase in parathyroid preservation with the use of ICGA (*p* < 0.01). Postoperative hypoparathyroidism on the first day (POD-1) showed a trend toward reduction in the ICGA group (25.8%) compared to the control group (35.8%), although this did not reach statistical significance (*p* = 0.09). The incidence of POD-1 hypocalcemia was similar between the groups, with 7.5% in the control group and 8.3% in the ICGA group (*p* = 0.81). No cases of permanent hypocalcemia were reported in either group. The operative time was found to be longer in the ICGA group, with a median of 90 min (range 50–450 min) compared to 70 min (range 40–360 min) in the control group, a difference that was statistically significant (*p* = 0.02) (Table 3). Subgroup analysis of patients who underwent central lymph node dissection showed that the rate of POD1 hypoparathyroidism was found to be decreased in the ICGA group (51.9% vs. 31.7%, *p* = 0.049) (Table 4).

## 4. Discussion

Thyroidectomy is one of the most frequently performed surgical procedures. Identification and preservation of parathyroid glands and their function is crucial to the success of this procedure. We hypothesized that the application of fluorescence imaging with ICGA during thyroidectomies could facilitate enhanced detection of the parathyroid glands and could subsequently assist surgeons in preserving the function of parathyroid glands. Our data indicate that the use of fluorescence imaging with ICGA resulted in a higher rate of parathyroid gland reimplantation and longer operative time. Moreover, the use of fluorescence imaging with ICGA showed a potential trend toward reduced incidence of postoperative hypoparathyroidism on day 1, although it did not reach statistical significance. 

Our study revealed that a significantly higher proportion of patients in the ICGA group underwent parathyroid gland autotransplantation compared to the conventional group (10% vs. 1.7%, *p* < 0.01). In a retrospective study by Rudin et al. [16] including 86 patients with ICGA compared to 120 without, the parathyroid autotransplantation rate was observed to be higher in the ICGA group (36% vs. 12%) like our study. This might have been caused by several factors. Decision-making by visual inspection only regarding parathyroid gland autotransplantation can pose significant challenges and its reliability has been questioned in multiple instances in the literature [17]. This can be the reason why surgeons, including those in our center, tend to avoid autotransplantation in gray zones by visual inspection only. However, ICGA can overcome this hesitation by offering a more accurate and objective measure of parathyroid gland perfusion, which has been reported by numerous authors [11,14,18]. Moreover, imaging of the specimen with a fluorescence camera enables the detection of inadvertently removed parathyroid glands and facilitates autotransplantation. Additionally, we have used a complementary step in our protocol different than the literature. A second injection of 1 mL of ICG was made if there was a suspicion of a hypoperfused parathyroid gland and the change in fluorescence intensity (FI) of the parathyroid gland was monitored. If there was an increase of more than 25% in the FI, we assumed that the vascularity of the gland was preserved and kept it. This technique provided us with an objective measurement and allowed us to eliminate the impact of surgeon experience and subjective evaluation like other studies in the literature [16,19]. In addition, measuring the change in FI after a second ICG injection instead of total FI can help to overcome a challenge of ICGA, which is the persistence of fluorescence within the parathyroid gland due to prior injection complicating the evaluation of current perfusion. This additional step was important and might contribute to the practice in the literature. 

This study showed a trend toward a decreased rate of POD1 hypoparathyroidism with the use of fluorescence imaging ICGA (35.8% vs. 25.8%, *p* = 0.09). Although the decrease in POD 1 hypoparathyroidism is not statistically significant, a subgroup analysis of patients who underwent central neck dissection revealed a significant decrease in POD1 hypoparathyroidism in the ICGA group (51.9% vs. 32.7%, *p* = 0.49). This improvement in functional preservation can be caused by easier protection of vascular supply and accurate assessment of perfusion and reimplantation of nonperfused glands. First, preserving blood supply is crucial for the functional preservation [20]. As shown in the literature, ICGA, after thyroid gland mobilization and before further dissection, allows intraoperative visualization of parathyroid gland vascular supply. Thus, it allows the surgeon to adjust dissection planes, taking care not to injure the parathyroid gland vascular supply which helps maintain function [19]. Secondly, we evaluated parathyroid perfusion in two different ways, which might improve the precision of detection and correct reimplantation minimizing hypoperfused glands to be left in place. First, we used objective measurement of parathyroid gland fluorescent intensity and compared it to a baseline level, which is the trachea. Then, glands suspected of hypoperfusion were assessed using a second injection and monitoring the increase in FI. A recent meta-analysis [21] showed that quantitative fluorescence intensity appeared to be more indicative of the level of tissue perfusion as compared to the visual score. Therefore, low perfusion according to FI could foresee postoperative hypoparathyroidism and help decision-making regarding autotransplantation. 

The improvement in early postoperative hypoparathyroidism did not translate into permanent hypoparathyroidism as the two groups were similar in terms of permanent hypoparathyroidism. Evaluating the impact of ICGA in decreasing the incidence of permanent hypoparathyroidism is problematic due to the low incidence of permanent hypoparathyroidism. The relatively rare occurrence of permanent hypoparathyroidism presents a significant challenge in demonstrating the effectiveness of ICGA or any intervention aimed at its prevention. The low incidence rate necessitates large sample sizes to achieve the statistical power required to detect a significant difference in outcomes between patient groups subjected to different surgical approaches. This is because the statistical analysis of uncommon events requires a broad dataset to distinguish between the natural variability of surgical outcomes and the actual effects of the intervention being studied. Consequently, studies with insufficient sample sizes may lack the sensitivity to capture subtle but clinically meaningful benefits of ICGA in reducing the rates of permanent hypoparathyroidism, potentially underestimating the value of this technological advancement in thyroid surgery. Therefore, a larger patient sample size would be necessary to accurately distinguish these differences. 

A drawback of ICGA fluorescence imaging is an increase in operative time by a mean of 24.5 min. Additional procedures in the ICGA group compared to the conventional group do not that take more than 10 min. Nevertheless, it should be considered that this group includes the cases where fluorescence imaging was used in our center for the first time. Hence, the difference can be explained by the inclusion initial phases learning curve and the time required for adjusting to new material. In addition, the maximum tissue depth near-infrared fluorescence can reach was reported to be 10 mm [22]. Considering this fact, surgeons might have opted for more meticulous dissection to minimize fatty tissue around the gland and optimize visualization. 

Limitations of this study include the retrospective design, the presence of two different surgeons, and the learning curve in the study process. Although propensity score matching was performed to adjust for confounding covariates such as age, sex, indication for surgery, preoperative calcium level, BMI, and presence of central neck dissection, which were known risk factors for postoperative hypoparathyroidism [23], it is important to underscore that despite the robustness of this method, it may not entirely eliminate the influence of all potential confounding covariates. Consequently, there may still be residual confounding that could influence the outcomes of our study. Moreover, another limitation of our study was the unequal sizes of the groups before applying propensity score matching. While this method helped to adjust for various confounding variables, ensuring a fair comparison between groups, the initial disparity in group sizes could introduce a subtle bias in our analysis. This disparity complicates the propensity score matching process, potentially leading to inadequate matches and the exclusion of participants, which can introduce selection bias. We acknowledge these aspects as constraints on both the robustness of our study’s methodology and the applicability of its conclusions.

Nonetheless, in the era of innovative perioperative imaging methods in all subspecialties of surgery [24,25], we will continue to use ICGA in our clinical practice, as we believe it provides a more precise prediction of parathyroid gland-viability especially in patients undergoing central neck dissection. This valuable information aids in decision-making for parathyroid autotransplantation and will give us an idea of which patients might be susceptible to hypoparathyroidism.

## 5. Conclusions

In conclusion, our study provides valuable insights into the role of fluorescence imaging using indocyanine green angiography and its potential to facilitate the preservation of parathyroid gland functionality. Our study indicates that the use of ICGA significantly increased parathyroid gland autotransplantation rates and showed a promising trend toward reduced incidence of postoperative hypoparathyroidism. Furthermore, our innovative technique of monitoring the change in fluorescence intensity after a second ICG injection has demonstrated its utility in eliminating subjective evaluation, offering a more objective assessment of parathyroid gland perfusion. This approach, along with the intraoperative visualization of the parathyroid vascular supply, offers surgeons a potentially valuable tool for preserving parathyroid function. Considering the potential benefits of ICGA, further large-scale, prospective studies are needed to evaluate its impact on rates of both transient and permanent postoperative hypoparathyroidism and to address the limitations observed in our study. Additionally, examining patient-specific factors may reveal how individual differences affect the success of ICGA in parathyroid gland preservation, ultimately enhancing surgical outcomes and patient care in thyroidectomy procedures.

## Figures and Tables

**Table 1:** Clinicopathological and Treatment Characteristics before Propensity Score Matching (ICGA: Indocyanine green angiography, BMI: Body mass index).

	Control (*n* = 175)	ICGA (*n* = 120)	*p*-Value
Gender			0.07
Female	132 (75.4%)	79 (65.8%)	
Male	43 (24.6%)	41 (34.2%)	
Age	49.14 ± 13.36	49.16 ± 13.13	0.99
BMI	27.3 ± 5.26	26.95 ± 5.36	0.58
Indication for Surgery			0.09
Malignancy	77 (44%)	65 (54.2%)	
Non-malignancy	98 (56%)	55 (45.8%)	
Preoperative Serum Calcium Level	9.58 ± 0.45	9.52 ± 0.43	0.24
Preoperative Serum Vitamin D Level	31 [23–38]	32 [20–39]	0.76
Central Neck Dissection	75 (42.9%)	49 (40.8%)	0.79

**Table 2:** Clinicopathological and Treatment Characteristics after Propensity Score Matching (ICGA: Indocyanine green angiography, BMI: Body mass index).

	Control (*n* = 120)	ICGA (*n* = 120)	*p*-Value
Gender			0.21
Female	88 (73.3%)	79 (65.8%)	
Male	32 (26.7%)	41 (34.2%)	
Age	48.69 ± 1.2	49.16 ± 13.1	0.78
BMI	26.87 ± 5.3	26.95 ± 5.36	0.91
Indication for Surgery			0.43
Malignancy	59 (49.2%)	65 (54.2%)	
Non-malignancy	61 (50.8%)	55 (45.8%)	
Preoperative Serum Calcium Level	9.6 [8.6–10.6]	9.6 [8.6–10.8]	0.21
Preoperative Serum Vitamin D Level	34 [22–41]	32 [20–39]	0.81
Central Neck Dissection	54 (45%)	49 (40.8%)	0.51

**Table 3:** Postoperative Outcomes after Propensity Score Matching (ICGA: Indocyanine green angiography, POD-1: Postoperative day 1).

	Control (*n* = 120)	ICGA (*n* = 120)	*p*-Value
Parathyroid Reimplantation	1 (0.8%)	12 (10%)	<0.01
POD-1 Hypoparahyroidism	43 (35.8%)	31 (25.8%)	0.09
POD-1 Hypocalcemia	9 (7.5%%)	10 (8.3%)	0.81
Permanent Hypocalcemia	0	0	0
Operative Time	70 [40–360]	90 [50–450]	0.02

**Table 4:** Rate of Hypocalcemia in Patients with Central Neck Dissection after Propensity Score Matching (ICGA: Indocyanine green angiography, POD-1: Postoperative day 1).

	Control (*n* = 54)	ICGA (*n* = 49)	*p*-Value
POD-1 Hypoparathyroidism	28 (51.9%)	16 (32.7%)	0.049

## Data Availability

The datasets analyzed during the current study are not publicly available due to privacy and ethical considerations. However, they are available from the corresponding author upon reasonable request.
